# Estimating tidal constituents in shallow waters from satellite altimetry using a 2D hydrodynamic model with nonlinear tide-surge interactions

**DOI:** 10.1007/s10236-025-01667-6

**Published:** 2025-03-03

**Authors:** Henrique Guarneri, M. Verlaan, D. C. Slobbe, F. Zijl, J. Pietrzak, L. Keyzer, Y. Afrasteh, R. Klees

**Affiliations:** 1 Delft University of Technology, P. O. Box 5048, 2600 GA Delft, The Netherlands; 2 Deltares, P. O. Box 177, 2600 MH Delft, The Netherlands

**Keywords:** 2D hydrodynamic model, Nonlinear tide-surge interaction, Satellite altimetry, Tidal harmonic analysis

## Abstract

Tidal models that incorporate satellite altimeter data have historically shown discrepancies in accuracy between shallow and deep marine environments. A recent study suggests that these differences may partly stem from neglecting the nonlinear tide-surge interactions in tidal analyses. In this study, we introduce a novel method for estimating tidal constituents from satellite altimeter data in shallow waters, leveraging a 2D hydrodynamic model that accounts for these nonlinear interactions. This approach substantially reduces the variance of unaccounted water level variability, thereby benefiting the estimation. A distinctive feature of our method is the treatment of prior model tidal constituents as stochastic, which helps manage the low temporal resolution of altimeter data by ensuring that unresolved tidal constituents are not updated. We tested our method in the data-rich northwest European continental shelf region, using the high-resolution 2D Dutch Continental Shelf Model version 7 (DCSM). Results show a substantial reduction in the standard deviations of residual water level time series in the shallow waters around Great Britain and in the German Bight, from 11 cm to 5 cm. In deep waters (>200 m), the median standard deviation decreased from 6.8 cm to 6.2 cm. When compared to state-of-the-art ocean tide and surge corrections from publicly available models, our method outperformed them in shallow waters (median standard deviation of 6.0 cm versus 7.5 cm), though the alternative products performed better in deep waters (median standard deviation of 5.5 cm versus 6.2 cm). An estimate of the accuracy at satellite crossovers resulted in an estimated total tidal error of about 1.5 cm (RSS VD). We acknowledge that comparisons in shallow waters are complicated, as alternative products do not account for nonlinear tide-surge interactions. Overall, the demonstration along-track tidal product developed in this study shows potential for improving the tidal representation in the DCSM model. In data-poor regions, the number of tidal constituents that can be reliably estimated using the method may be limited, and alternative strategies might be needed to evaluate the model’s uncertainty in representing tides.

## Introduction

Satellite altimetry offers valuable data for the calibration and validation of hydrodynamic models, even in regions rich in tide gauge data, such as the northwest European continental shelf. Its key advantage is the broad spatial coverage it provides: while tide gauges are mostly confined to the coast, altimeter tracks span a large part of the global oceans. Despite their potential, altimeter data have seen limited use in developing the operational models for water level forecasting in Dutch coastal waters. The reluctance arises from the challenges posed by the complex hydrodynamics of shallow Dutch waters and the low temporal resolution of altimeter data—the best sampling frequency is 9.915642 days for the TOPEX/Poseidon and Jason (TPJ) satellites, except at crossover points. These challenges apply to other shallow water regions as well. The overall objective of our research is to unlock the potential of satellite altimetry for calibrating and validating hydrodynamic models of shallow waters, focusing specifically on the representation of the tides.

Estimating tidal constituents from satellite altimeter data in shallow waters is inherently challenging. Stammer et al. (2014) showed that the root-sum-square (RSS) differences between tide observations and the best models for eight major constituents increase from 0.9 cm in pelagic regions to 5.0 cm in shelf areas and 6.5 cm in coastal regions. Previous studies (e.g., Andersen 1999; Andersen et al. 2006; Ray et al. 2011) have examined the causes of these discrepancies, which are summarized in Guarneri et al. (2023). One major, previously overlooked cause identified by Guarneri et al. (2023) is the omission of nonlinear tide-surge interactions when removing tide and surge estimates from altimeter-derived water levels before conducting tidal analysis. These interactions, documented in numerous locations globally (e.g., Proudman 1955b, a; Prandle and Wolf 1978; Johns et al. 1985; Horsburgh and Wilson 2007; Zhang et al. 2010; Idier et al. 2012, 2019; Arns et al. 2020), alter the phase of tidal signals while the tides themselves modulate non-tidal signals.

The study by Guarneri et al. (2023) used partially synthetic satellite altimeter time series generated from the Europlatform tide gauge record located in the southern North Sea, tide-surge water level time series from the 2D Dutch Continental Shelf Model - Flexible Mesh (DCSM), and tidal *and* surge water level time series obtained using the DCSM, FES2014 (Lyard et al. 2021) and the Dynamic Atmospheric Correction (DAC – Carrère et al. 2011) product. Here, the DCSM tide-surge water levels include the nonlinear tide-surge interactions. Their results showed that removing nonlinear tide-surge interactions reduced RSS differences for the eight main tidal constituents by more than 50% compared to removing the sum of DCSM tidal and DCSM surge water levels, or FES2014 tidal and DAC surge water levels. This finding highlights the potential to improve coastal tidal estimation from satellite altimeter data. Building on Guarneri et al. (2023), this study demonstrates these improvements using *real* satellite altimeter data.

However, this task is not straightforward because of additional complexities. The nonlinear dynamics in shallow waters cause compound and overtides (also called shallow water tides), and dampening of weaker constituents in the presence of the tidal currents of stronger constituents (Ray et al. 2011). Estimating these shallow water constituents requires much more data than in linear regimes. The low temporal resolution of altimeter time series, along with their limited length (about 30 years for the TPJ satellite series), makes it unlikely that all constituents can be reliably estimated. Further challenges include i) the small amplitude of most shallow water constituents in absolute sense and/or compared to the nontidal signals in the altimeter data, ii) the lower accuracy of altimeter data in coastal waters compared to deep waters (e.g., Gommenginger et al. 2011; Vignudelli et al. 2011; Passaro et al. 2014; Vignudelli et al. 2019), and iii) limitations of equilibrium tide theory for nodal corrections in shallow waters (e.g., Hagen et al. 2021). According to Andersen (1999), the first issue is the limiting factor that determines which shallow water constituents can be resolved from altimeter data. Regarding the second issue it is worth mentioning that over the last decade major advances have been made; we refer to Laignel et al. (2023) for a recent review. These improved data also contributed to an improved estimation of coastal tidal constituents (e.g., Piccioni et al. 2018, 2021; Hart-Davis et al. 2021a; Seifi and Filmer 2023).

We expect that incorporating nonlinear tide-surge interactions before conducting tidal harmonic analysis will enable the reliable extraction of more shallow water constituents from altimeter data, though not ‘all’ and not everywhere. The low temporal resolution, limited length of the satellite record, and the small amplitudes of most shallow water constituents still impose constraints. Developing a purely satellite-derived tidal product would require determining, for each location, which tidal frequencies contain energy, which constituents can be directly estimated and which must be inferred (e.g., Foreman and Henry 1989; Egbert and Ray 2017; Ray 2017) from the major tidal constituents. Such an approach, which would result in a tidal product comprising a spatially varying set of constituents, does not appear to be current practice. Global ocean tide models including TPXO9 (Egbert and Erofeeva 2002), GOT410 (Ray 1999), DTU10 (Cheng and Andersen 2011), FES2022b (LEGOS et al. 2024), and EOT20 (Hart-Davis et al. 2021a) all provide a fixed limited set of constituents, i.e., the set contains at most a few dozen constituents and is the same everywhere. Besides, there seems no consensus on which constituents should be estimated directly and which should be inferred (Ray 2017; Karbon et al. 2018).

The main objective of this paper is to develop a procedure for generating an along-track tidal product from satellite altimeter data that exploits a 2D hydrodynamic model accounting for the nonlinear tide-surge interactions. We demonstrate this procedure using TPJ altimeter data for the northwest European continental shelf, validate the obtained along-track tidal product, and assess its potential for future hydrodynamic model calibration through data assimilation. The procedure avoids the need to pre-select which tidal constituents to estimate from altimeter data by treating the model-derived tidal constituents as stochastic. Altimeter data contribute to the constituent estimates based on their precision relative to the model’s precision, resulting in a combined altimeter-model product. While this approach is known in other fields (e.g., Lahoz and Schneider 2014; Lewis et al. 2006), it has not been applied to tidal harmonic analysis. Essentially, it resembles the approach used in developing assimilative tide models, with the difference that the combination is done a-posteriori. The resulting product will be the first that is generated by explicitly accounting for the nonlinear tide-surge interactions. We do not aim to produce the best possible tidal product. Various simplifications are used. These include: i) usage of a fixed set of tidal constituents for the entire domain covered by the hydrodynamic model; and ii) reliance on equilibrium tide theory in computing the nodal corrections. In developing the procedure, we take advantage of the fact that in the northwest European continental shelf a huge number of tide gauges is available and that we have access to a high-resolution hydrodynamic model. To what extent the procedure is applicable elsewhere is not part of this study. Validation is performed using the standard deviation of residual water levels, following a *K*-fold cross-validation strategy (Bengio and Grandvalet 2003). We compare the relative error in M2 phase and amplitude at coastal tide gauges and nearby altimeter data locations to assess the potential of the along-track tidal product for calibrating the hydrodynamic model through data assimilation.

The paper is organized as follows: Sect. 2 describes the proposed procedure for generating the along-track tidal product, its implementation for the northwest European continental shelf, and the validation methods. Section 3 introduces the datasets and models used. Results are presented and discussed in Sect. 4. Finally, we conclude by summarizing the main findings of the paper in Sect. 5.

## Methods

### Procedure to generate the along-track tidal product

Following the notation established in Guarneri et al. (2023), we denote the water level by *h*. The sub- and superscripts specify what part of the water level is referred to (i.e., total, tide, surge, or tide-surge) and the source from which it is obtained (sat (satellite-derived) or mod (model-derived)). The satellite-derived water levels ${h}^{\text {sat}}$ are available at times ${t}^{\text {sat}}$, while the model-derived water levels ${h}^{\text {mod}}$ are available at times ${t}^{\text {mod}}$. For the TPJ satellite altimeter data being used in this study, times ${t}^{\text {sat}}$ form a time series covering the period 1993 through 2017 with a sampling interval of 9.915642 days. Note, however, that ${t}^{\text {sat}}$ varies per location, and the time series may contain gaps. Model-derived time series cover the same period but are sampled every 10 minutes. ${h}^{\text {mod}}({t}^{\text {sat}})$ referred to below is obtained by interpolating from ${h}^{\text {mod}}({t}^{\text {mod}})$. The hat ( $\hat{\phantom{0}}$ ) and tilde ( $\tilde{\phantom{0}}$ ) indicate the set of amplitudes and phases estimated from ${h}({t}^{\text {mod}})$ and ${h}({t}^{\text {sat}})$, respectively.

The tidal analysis is based on harmonic analysis of the *residual* water level time series, meaning that, before estimation, we remove the water levels obtained from a background (or prior) model. In this study, the prior model is a 2D hydrodynamic model that accounts for nonlinear tide-surge interactions. However, the residual water levels may still contain any unmodeled or mismodeled tidal water level variability. The observation equation (in complex form) reads:

1 $$\begin{aligned} {h}_{\text {total}}^{\text {sat}}({t}^{\text {sat}})&-{h}_{\text {tide-surge}}^{\text {mod}}({t}^{\text {sat}})&= \sum _{l=1}^L \left( \delta \!A_{l}\ e^{i\ \delta \phi _ {l}}\right) \left( f_{l}({t}^{\text {sat}}) e^{iu_ {l}({t}^{\text {sat}})}\right) \nonumber \\&\quad e^{iv_{l}({t}^{\text {sat}})} + \eta , \end{aligned}$$

where *L* is the total number of constituents being estimated, $\left( \delta \!A_{l}\ e^{i\ \delta \phi _ {l}}\right)$ is the unknown complex residual amplitude-phase pair, $f_{l}({t}^{\text {sat}}) e^{iu_ {l}({t}^{\text {sat}})}$ is the nodal correction, $e^{iv_{l}({t}^{\text {sat}})}$ is the phase of the equilibrium tide, and $\eta$ the residual. Equation 1 can be written in matrix–vector form as:

2 $$\begin{aligned} \textbf{y}&= \textbf{A}\textbf{x}+\varvec{\eta }, \end{aligned}$$

where $\textbf{y}$ is the $m\times 1$ vector of residual water levels, $\textbf{A}$ is the $m\times L$ design matrix, $\textbf{x}$ is the $L\times 1$ vector with unknown complex residual amplitude-phase pairs, and $\varvec{\eta }$ is the $m\times 1$ vector with residuals. The stochastic properties of the residuals are described by the stochastic model

3 $$\begin{aligned} E\left\{ \varvec{\eta }\right\} = 0,\,\, E\left\{ \varvec{\eta }\varvec{\eta }^ {\textsf{T}}\right\} =D\left\{ \varvec{\eta }\right\} =\textbf{Q}_{{h}_{\text {total}}^{\text {sat}}}, \end{aligned}$$

where $E\left\{ \cdot \right\}$ denotes the expectation operator, $D\left\{ \cdot \right\}$ the dispersion operator, and $\textbf{Q}_{{h}_{\text {total}}^{\text {sat}}}$ is the variance-covariance matrix of the satellite-derived water levels. For the TPJ data being used in this study, we assume

4 $$\begin{aligned} \textbf{Q}_{{h}_{\text {total}}^{\text {sat}}} = \sigma ^2 \textbf{I}, \end{aligned}$$

where $\sigma$ is the standard deviation of the uncertainty in the TPJ-derived sea surface heights plus the uncertainty due to unmodeled or mismodeled nontidal water level variability, and $\textbf{I}$ is the identity matrix.

**Table 1:** Complete set of tidal constituents as defined by Rijkswaterstaat, the Dutch governmental agency for public works and water management

Category	Constituents
Long-period	SA, SM
Diurnal	Q1, O1, M1C, P1, S1, K1
Semi-diurnal	3MKS2, 3MS2, OQ2, MNS2, 2ML2S2, NLK2, MU2, N2, NU2
	MSK2, MPS2, M2, MSP2, MKS2, LABDA2, 2MN2, T2, S2
	K2, MSN2, 2SM2, SKM2
Third-diurnal	NO3, 2MK3, 2MP3, SO3, MK3, SK3
Fourth-diurnal	4MS4, 2MNS4, 3MS4, MN4, 2MLS4, 2MSK4, M4, 3MN4, MS4
	MK4, 2MSN4, S4
Fifth-diurnal	**MNO5**, 3MK5, 2MP5, 3MO5, MSK5, 3KM5
Sixth-diurnal	3MNS6, 2NM6, 4MS6, 2MN6, 2MNU6, 3MSK6, M6, MSN6
	MKNU6, 2MS6, 2MK6, 3MSN6, 2SM6, MSK6
Seventh-diurnal	2MNO7, M7, 2MSO7
Eighth-diurnal	2(MN)8, 3MN8, M8, 2MSN8, 2MNK8, 3MS8, 3MK8, 2(MS)8
	2MSK8
Ninth-diurnal	3MNK9, 4MK9, 3MSK9
Tenth-diurnal	4MN10, M10, 3MSN10, 4MS10, 2(MS)N10, 3M2S10
Eleventh-diurnal	4MSK11
Twelfth-diurnal	M12, 4MSN12, 5MS12, 4M2S12

The constituent MNO5 is not estimated because its alias period is too close to the one of 2ML2S2

To obtain the full amplitudes and phases, we restore those estimated from ${h}_{\text {tide-surge}}^{\text {mod}}({t}^{\text {mod}})$, i.e., we add $\hat{h}^{\text {mod}}$. The main feature of our approach is treating $\hat{h}^{\text {mod}}$ as stochastic. The degree to which a particular constituent is ‘updated’ based on satellite data depends on the relative precision of the satellite-derived estimate compared to that of the model-derived estimate. Since the satellite- and model-derived time series are independent, this leads to the well-known weighted least-squares solution (e.g., Lewis et al. 2006):

5 $$\begin{aligned} \tilde{\textbf{x}}^{\text {sat/mod}}&= \hat{\textbf{x}}^{\text {mod}} + \textbf{Q}_{\hat{\textbf{x}}^{\text {mod}}}\textbf{A}^{\textsf{T}}\left( \textbf{Q}_{{h}_{\text {total}}^{\text {sat}}} + \textbf{A}\textbf{Q}_{\hat{\textbf{x}}^{\text {mod}}}\textbf{A}^{\textsf{T}}\right) ^{-1} \textbf{y}, \end{aligned}$$

where $\tilde{\textbf{x}}^{\text {sat/mod}}$ is the vector of unknown complex amplitude-phase pairs, $\hat{\textbf{x}}^{\text {mod}}$ is the vector of complex amplitude-phase pairs estimated from ${h}_{\text {tide-surge}}^{\text {mod}}({t}^{\text {mod}})$ (i.e., the complex equivalent of $\hat{h}^{\text {mod}}$), and $\textbf{Q}_{\hat{\textbf{x}}^{\text {mod}}}$ is the associated variance-covariance matrix. The resulting set of amplitudes and phases, $\tilde{h}^{\text {sat/mod}}$ is a combined satellite- and model-derived product. The second term on the right-hand side of Eq. 5 represents the update based on the satellite data. We refer to this contribution, also described as the residual amplitudes and phases, as $\delta \tilde{h}^{\text {sat}}$. The corresponding *residual* tidal water levels are denoted as $\delta {h}_{\text {tide}}^{\text {sat}}$.

$\textbf{Q}_{\hat{\textbf{x}}^{\text {mod}}}$ is assumed to be a diagonal matrix, i.e., error correlations among the tidal coefficients are ignored. The variances can be obtained from the differences between tidal constituents estimated from tide gauge records and model-derived ones. Doing so, requires multiple tide gauges that are properly distributed over the area of interest. To quantify the differences we use the vector differences (VDs). For a specific constituent, the VD is defined as (Le Provost et al. 1995):

6 $$\begin{aligned} \text {VD} = \sqrt{(A^{\text {TG}} \cos {g^{\text {TG}}} - A^{\text {mod}} \cos g^{\text {mod}})^2 + (A^{\text {TG}} \sin g^{\text {TG}} - A^{\text {mod}} \sin g^{\text {mod}})^2}, \end{aligned}$$

where *A* and *g* represent the amplitude and phase, and the superscripts ‘TG’ and ‘mod’ indicate the source from which they are obtained; tide gauge (TG) or model. Per constituent we obtain *J* vector differences, where *J* equals the number of tide gauges. From these we compute the variance. To build $\textbf{Q}_{\hat{\textbf{x}}^{\text {mod}}}$, we split the variance equally over the real and complex part of the amplitude-phase pair.

**Fig. 1:**
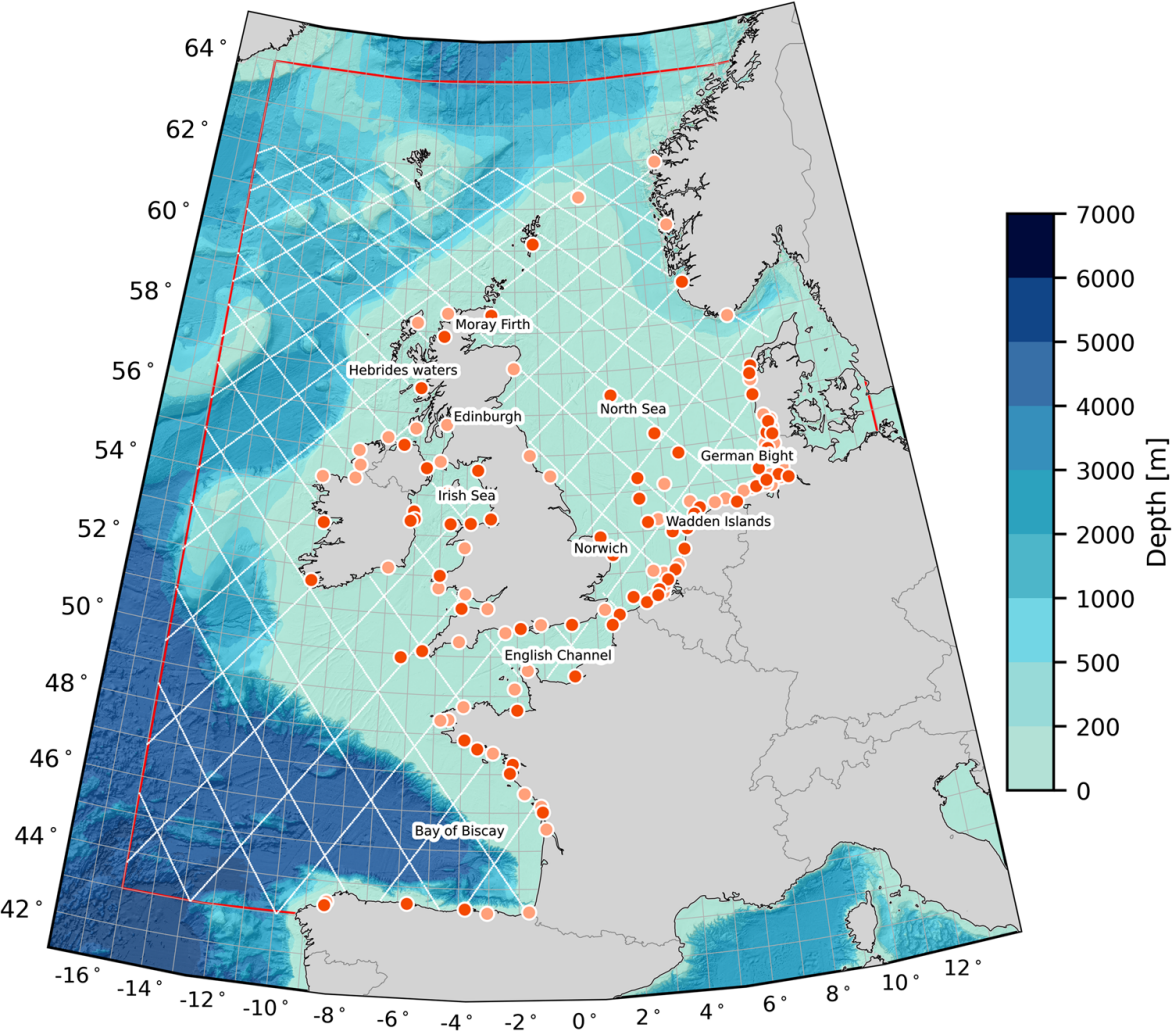
Map of the northwest European continental shelf that defines our area of interest. The area depicted by the red polygon corresponds to the domain covered by the 2D Dutch Continental Shelf Model - Flexible Mesh (see Sect. 3.2). The map also shows the locations of the tide gauges used to determine $\textbf{Q}_{\hat{\textbf{x}}^{\text {mod}}}$ and the X-TRACK TPJ tracks (white lines) used in the analysis. The orange and red subsets (including 69 and 68 tide gauges, respectively) have been used in the sensitivity analysis presented in Sect. 4.1.2. In the background, we show the GEBCO_2022 bathymetry (GEBCO Compilation Group 2022)

### Implementation of the method for the northwest European continental shelf

The method described in the previous section is applied to obtain an along-track tidal product for the northwest European continental shelf. The following considerations are made in this implementation:

1. Radiational tides (tides caused by atmospheric conditions and solar heating) are considered part of the tidal signal. Consequently, we restore the contributions of the removed surge signal at tidal frequencies.
2. To represent the total tidal water level across the northwest European continental shelf, we use the mean water level and 93 tidal constituents (listed in Table 1) as defined by Rijkswaterstaat, the Dutch governmental agency for public works and water management. The original set included the MNO5 constituent, which we excluded due to its alias period being too close to that of 2ML2S2. This set of constituents is the standard used in the Netherlands for tidal harmonic analysis of tide gauge records with a minimum length of one year. While it may lack some constituents that are significant in other regions (and vice versa), the impact of not using a regionally-tailored set outside Dutch waters is expected to be minimal. This is because the tidal harmonic analysis is applied to the *residual* water levels, obtained by subtracting the tide-surge water levels from the DCSM model. Most of the missing constituents, such as Ssa, Msm, Mm, Msf, Mf, 2Q1, SIG1, J1, 2N2, M3, and MO3, are already accounted for in the DCSM tide-surge water levels and removed to the extent the model represents them prior to the analysis.
3. $\sigma$, used to define $\textbf{Q}_{{h}_{\text {total}}^{\text {sat}}}$ is set at 6 cm. It includes the uncertainty in the altimeter data and any corrections applied to compute the sea surface heights, as well as the uncertainty due to unmodeled or mismodeled nontidal water levels. Based on the literature, we estimate that the uncertainty in sea surface heights is around 3 cm (Ponte et al. 2007). We have no reference for the second contribution. The standard deviations of the differences between TPJ-derived and model-derived water levels ranges from approximately 5 to 13 cm (see Fig. 5b). Since this also contains errors in the model-derived tidal water levels, we adopt a value close to the lower bound of this range. The sensitivity of the solution to the magnitude of $\sigma$ is explored in Sect. 4.1.1.
4. The *J* tide gauges used to obtain $\textbf{Q}_{\hat{\textbf{x}}^{\text {mod}}}$ are shown in Fig. 1. The sensitivity of $\tilde{h}^{\text {sat/mod}}$ to the set of tide gauges used to determine $\textbf{Q}_{\hat{\textbf{x}}^{\text {mod}}}$ is addressed in Sect. 4.1.2.
5. Tidal analyses are conducted using our extended version of the HATYAN package (Veenstra 2022), an open-source, Python-based software package for tidal harmonic analysis and prediction, owned by Rijkswaterstaat and developed and maintained by Deltares. The software applies nodal corrections that are based on the equilibrium tide theory, which is known to be less accurate in shallow waters (e.g., Ku et al. 1985; Hagen et al. 2021). However, we believe the impact of this simplification is small because: i) the tidal analysis is applied to *residual* water levels after removing the tide-surge water levels from the DCSM model, which inherently includes nodal oscillations and their shallow-water modulations, and ii) the analysis period covers more than a full nodal cycle (18.6 years), allowing any errors due to nodal modulation to average out over time.

### Metrics used to present and validate the solution and to assess its potential for improving hydrodynamic models

#### Root-sum-square of the vector differences

To quantify the contribution of altimeter data to $\tilde{h}^{\text {sat/mod}}$, we calculate the root-sum-square (RSS) of the VDs for all 93 tidal constituents, as well as the RSS values of the VDs for specific categories of constituents (as shown in Table 1). The RSS is commonly used to summarize performance for multiple tidal constituents, and it is computed as follows:

7 $$\begin{aligned} \text {RSS} = \sqrt{\sum _{i=1}^{N}{(A^{\text {sat/mod}}_{l} \cos {g^{\text {sat/mod}}_{l}} - A^{\text {mod}}_{l} \cos g^{\text {mod}}_{l})^2 + (A^{\text {sat/mod}}_{l} \sin g^{\text {sat/mod}}_{l} - A^{\text {mod}}_{l} \sin g^{\text {mod}}_{l})^2}}, \end{aligned}$$

where *N* represents the total number of tidal constituents included in the summation. To evaluate the sensitivity of $\tilde{h}^{\text {sat/mod}}$ to i) the magnitude of $\sigma$ (see Eq. 4) and ii) the set of tide gauges used to determine $\textbf{Q}_{\hat{\textbf{x}}^{\text {mod}}}$, we use the RSS of the VDs of all 93 constituents, with $\tilde{h}^{\text {sat/mod}}$ serving as the reference.

#### Standard deviation of the residual water levels

To validate the along-track tidal product for the northwest European continental shelf, we compare the standard deviations (SDs) of the residual water levels obtained as ${h}_{\text {total}}^{\text {sat}}({t}^{\text {sat}})-{h}_{\text {tide-surge}}^{\text {mod}}({t}^{\text {sat}})$ to those obtained as ${h}_{\text {total}}^{\text {sat}}({t}^{\text {sat}})-{h}_{\text {tide-surge}}^{\text {mod}}({t}^{\text {sat}})-\delta {h}_{\text {tide}}^{\text {sat}}({t}^{\text {sat}})$. There is a risk that our tidal parametrization will capture some of the nontidal water level variability (which is not specific to our approach but is always the case for tidal harmonic analysis). This results in lower SDs but is by no means indicative of a better quality of our tidal product. To address this, we compute the SDs of the residual time series obtained as ${h}_{\text {total}}^{\text {sat}}({t}^{\text {sat}})-{h}_{\text {tide-surge}}^{\text {mod}}({t}^{\text {sat}})-\delta {h}_{\text {tide}}^{\text {sat}}({t}^{\text {sat}})$ using a *K*-fold cross-validation strategy. In this strategy, we split the dataset into *K* training and validation sets. Each validation dataset covers three consecutive years. The periods do not overlap; i.e., the first period covers the first three years of the total measurement time span, the second period covers the second three years, and so on. All data outside each validation period form the training set. The TPJ dataset, covering 1993 to 2017, yields $K = 8$ (with 2017 excluded from validation). A tidal harmonic analysis is conducted on each training set, and the resulting tidal estimates are used to construct the residual tidal water level time series ($\delta {h}_{\text {tide}}^{\text {sat}}({t}^{\text {sat}})$) for the corresponding validation period. The complete $\delta {h}_{\text {tide}}^{\text {sat}}({t}^{\text {sat}})$ time series then allows us to compute the SDs. Here, SDs are computed as $1.4826\ \times$ the median absolute deviation (Cook and Weisberg 1982; Rousseeuw and Croux 1993) to reduce the impact of outliers.

To evaluate our product’s performance, we not only compare with the SDs of the residual time series obtained by ${h}_{\text {total}}^{\text {sat}}({t}^{\text {sat}})-{h}_{\text {tide-surge}}^{\text {mod}}({t}^{\text {sat}})$. We also look at the X-TRACK tidal product. Additionally, we investigate whether our product’s quality can be determined without correcting for surge and tide-surge interactions. The complete overview of all sets of residual water level time series considered in the validation is provided in Table 2. The comparisons are organized into three groups. In the first group, $\xi$ with superscripts 1 and 2 represents the SLAs derived from the removal of tide, surge, and tide-surge interactions. In the second group, $\zeta$ with superscripts 1 and 2 represent the SLAs with only tide and surge removed and serve the comparisons with X-TRACK and the FES2022b tidal model. It should be noted that differences in the surge water level from DCSM and DAC may impact the comparison between $\zeta$ and $\xi$. Lastly, to isolate the impact of the tide correction ($\delta {h}_{\text {tide}}^{\text {sat}}({t}^{\text {sat}})$), we use $\varsigma$ with superscripts 1 to 3, which excludes corrections for surge and tide-surge interaction. Doing so incurs higher residuals, which may obscure certain differences.

**Table 2:** Overview of the different sets of residual water level time series considered in the validation presented in Sect. 4.2

Label	Definition
$\xi ^{(1)}$	${h}_{\text {total}}^{\text {sat}}({t}^{\text {sat}})-{h}_{\text {tide-surge}}^{\text {mod}}({t}^{\text {sat}})-\delta {h}_{\text {tide}}^{\text {sat}}({t}^{\text {sat}})$
$\xi ^{(2)}$	${h}_{\text {total}}^{\text {sat}}({t}^{\text {sat}})-{h}_{\text {tide-surge}}^{\text {mod}}({t}^{\text {sat}})$
$\zeta ^{(1)}$	${h}_{\text {total}}^{\text {sat}}({t}^{\text {sat}})-{h}_{\text {tide}}^{\text {X-TRACK}}({t}^{\text {sat}})-{h}_{\text {surge}}^{\text {DAC}}({t}^{\text {sat}})$
$\zeta ^{(2)}$	${h}_{\text {total}}^{\text {sat}}({t}^{\text {sat}})-{h}_{\text {tide}}^{\text {FES2022b}}({t}^{\text {sat}})-{h}_{\text {surge}}^{\text {DAC}}({t}^{\text {sat}})$
$\varsigma ^{(1)}$ $^{*1}$	${h}_{\text {total}}^{\text {sat}}({t}^{\text {sat}})-{h}_{\text {tide}}^{\text {mod}}({t}^{\text {sat}})-\delta {h}_{\text {tide}}^{\text {sat}}({t}^{\text {sat}})$
$\varsigma ^{(2)}$ $^{*1}$	${h}_{\text {total}}^{\text {sat}}({t}^{\text {sat}})-{h}_{\text {tide}}^{\text {mod}}({t}^{\text {sat}})$
$\varsigma ^{(3)}$	${h}_{\text {total}}^{\text {sat}}({t}^{\text {sat}})-{h}_{\text {tide}}^{\text {X-TRACK}}({t}^{\text {sat}})$
$^{*1}$ ${h}_{\text {tide}}^{\text {mod}}$(${t}^{\text {sat}}$) is computed from a synthesis of $\hat{h}^{\text {mod}}$	

Note that ‘mod’ in this study always refers to the DCSM model (see Sect. 3.2)

#### M2, S2, K1, and O1 amplitude and phase error patterns

To assess the potential of our new tidal product for future hydrodynamic model calibration by means of data assimilation, we compare the errors in the model-derived amplitudes and phases for the M2, S2, K1, and O1 constituents at tide gauge locations with our estimated residual amplitudes and phases at the TPJ data points. We expect model errors in the amplitudes and phases at the tide gauge locations to align with the residual amplitudes and phases at nearby TPJ data points. While this analysis can be extended to all tidal constituents, we focus here on the four main constituents M2, S2, K1, and O1. The analysis includes all tide gauges shown in Fig. 1. Instead of comparing absolute values, we normalize these by the local model-derived amplitudes and phases.

## Data

A number of observation and model-derived datasets were used in this study. This section briefly introduces each of them.

### X-TRACK along-track sea level anomalies and tidal constants

The X-TRACK along-track SLAs are produced with the X-TRACK processing system (Birol et al. 2017) developed by the Center of Topography of the Ocean and Hydrosphere (CTOH) in Toulouse. It uses as input the measurements and parameters provided in the geophysical data records plus additional corrections and auxiliary data specifically distributed by the CTOH. The product was created to improve the completeness and quality of sea surface height information received from satellite altimetry in coastal waters. The SLAs have been projected onto reference tracks with a point separation of about 6–7 km. In this study, we used the TPJ data from February 1993 to January 2018 of version 2017 (doi 10.6096/CTOH_X-TRACK_2017_02). The use of version 2017 ensures consistency with the X-TRACK tidal constants product used as a reference in Sect. 4.2.

The 73 X-TRACK tidal constants (CTOH/LEGOS France 2018) were obtained by means of harmonic analysis from the X-TRACK SLA data for TOPEX/Poseidon interleaved & Jason-1 interleaved, and TOPEX/Poseidon & Jason-1 & Jason-2. The time series start at Feb 28, 1993 and ends at July 24, 2015. The data and processing are briefly described in CNES/LEGOS/CTOH (2020). The SLAs from which the residual tidal constituents were estimated, were obtained by removing the FES2012 tidal water level and the surge water level obtained from the DAC. Both corrections are included in the SLA product.

### Tide-surge water levels from the DCSM

The 2D Dutch Continental Shelf Model - Flexible Mesh (DCSM; Zijl et al. (2022)) is the successor to the version described in Zijl et al. (2013, 2015). The model simulates the tide-surge water level variability across the northwest European continental shelf, spanning $15^\circ$W to $13^\circ$E and $43^\circ$N to $64^\circ$N, by solving the depth-integrated shallow water equations for hydrodynamic modeling of free-surface flows (Leendertse 1967; Stelling 1984). The model incorporates nonlinear tide-surge interactions and is implemented using the Delft3D Flexible Mesh Suite (or D-HYDRO Suite), allowing for unstructured grids. The grid resolution increases with decreasing water depth: in deep waters, the resolution is 4.9–8.1 km (depending on latitude) in the east–west direction and 7.4 km in the north–south direction, while over the continental shelf, it increases from 2.5–4.1 km $\times$ 3.7 km to $840 \times 930$ m in Dutch waters. Overall, the resolution is higher than that of the model used by Horsburgh and Wilson (2007) to study tide-surge interactions (they used a model with a resolution of 12 km), providing confidence that the DCSM can capture these interactions at the spatial scales resolvable by the model.

To reduce uncertainty in bottom roughness, an automated calibration using the ‘Doesn’t Use Derivative’ algorithm (Ralston and Jennrich 1978) has been performed. In doing so, all 2017 data from 195 tide gauges were used. Here, extra weight in the cost function has been given to the Dutch coastal tide gauges, since the model is primarily intended to obtain an accurate water level representation in Dutch coastal areas.

Water level boundary conditions are applied at the northern, western, and southern open boundaries. They are the sum of the astronomical tidal water levels and the surge. The tidal water levels are obtained from a harmonic expansion of 31 tidal constituents (i.e., 2N2, J1, K1, K2, LABDA2, L2, M2, M3, M4, M6, M8, MF, MFM, MM, MN4, MNS2, MS4, MSF, MU2, N2, N4, NU2, O1, P1, Q1, R2, S1, S2, S4, SSA, T2) retrieved from the global ocean tide model FES2012 (Carrère et al. 2013) supplemented with the solar annual Sa constituent obtained from an earlier version of the model. The surge at the open boundaries is approximated by a time- and space-dependent inverse barometer correction (see Zijl et al. 2022, Sect 2.6.2). In our simulations, the tidal potential representing the direct body force of the gravitational attraction of the moon and sun on the mass of water has been switched on. The time- and space-varying atmospheric wind and pressure forcings are obtained from the ECMWF’s ERA5 reanalysis dataset (Hersbach et al. 2020).

Zijl et al. (2022) validated the model against tide gauge data from both Dutch coastal and offshore locations. For its tidal representation, the average root-mean-square error (RMSE) is 3.6 cm for offshore gauges and 4.5 cm for coastal gauges. For the surge, the corresponding RMSE values are 4.2 cm and 5.3 cm, respectively. When considering total water levels (tide $+$ surge), the RMSE increases to 5.5 cm offshore and 6.9 cm at coastal stations. The RMSEs obtained for the 3D DCSM-FM model (Zijl et al. 2022) are at best 0.7 cm lower. Validation outside of Dutch waters was not conducted, though model performance is anticipated to be lower in these regions.

At X-TRACK altimeter data locations, tide-surge water levels were linearly interpolated in space and time from the 10-minute sampled time series at three to five surrounding model grid points. A sanity check (not included in this paper) confirmed that the interpolation errors are negligible for this application.

**Fig. 2:**
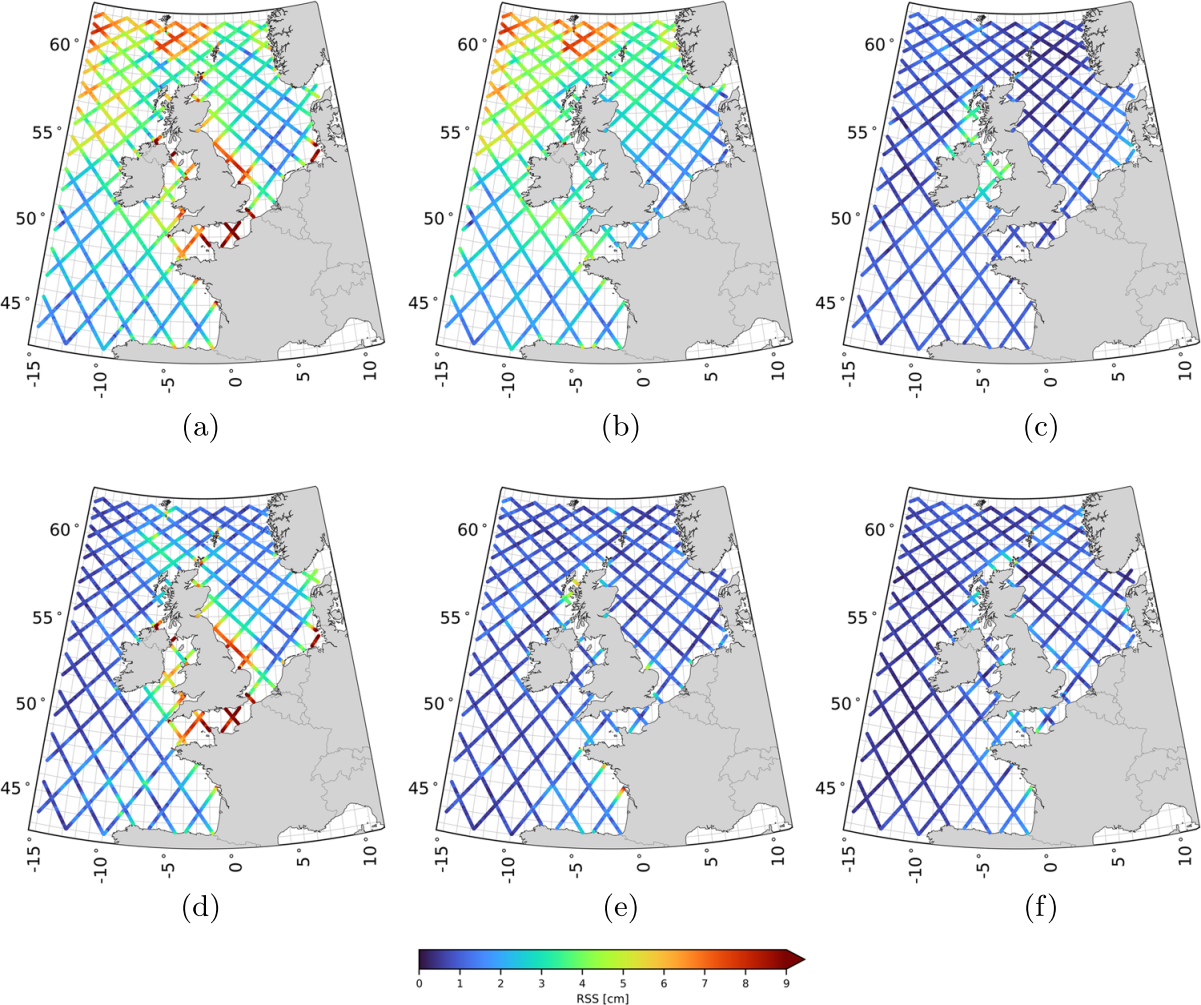
RSS values of the VDs for all 93 constituents (a), the long-period constituents (b), the diurnal constituents (c), the semi-diurnal constituents (d), the fourth-diurnal constituents (e), and the sixth-diurnal constituents (f)

### FES2022b tidal water levels

The FES2022b model is the most recent publicly available version of the finite element solution tidal model (Carrère et al. 2013). It is a so-called ‘assimilative model’, i.e., the atlas is computed using a hydrodynamic model that is coupled to a data assimilation code. The data being assimilated include altimeter data from various satellite missions and tide gauges. The atlas includes 34 tidal constituents that are provided on a regular 2 min. grid resolution. For a more detailed description of FES2022b, we refer to LEGOS et al. (2024).

### Surge water levels from the Dynamic Atmospheric Correction product

The dynamic atmospheric correction is a gridded product delivered by Aviso+ (LEGOS/CNRS/CLS 1992). The 6-hourly, $0.25^{\circ } \times 0.25^{\circ }$ grids describe the ocean response to atmospheric wind and pressure forcing computed using the Mog2D barotropic model (Carrère and Lyard 2003; Carrère et al. 2016) for high frequencies (i.e., less than 20 days), and the inverted barometer correction (Ponte 2006) for lower frequencies.

In our analyzes, we used the DAC corrections included in the X-TRACK along-track SLA product.

### Tide gauge records

Tide gauge records were sourced from an internal Deltares database, with most records acquired in coastal waters (see Fig. 1). These records were originally obtained from local authorities responsible for operating the tide gauges. Outliers were removed from all records, and small gaps (less than one hour) were filled using linear interpolation. For computing $\textbf{Q}_{\hat{\textbf{x}}^{\text {mod}}}$, we included all tide gauges records that: i) contain at least five consecutive years of data within the period 1993–2017; and ii) have an RSS of the VDs for all 93 constituents (see Eq. 6) less than or equal to 20 cm. Note that $A^{\text {mod}}$ and $g^{\text {mod}}$ were obtained from a model run covering the entire period (1993–2017). A total of 137 tide gauge records met these criteria. To assess the sensitivity of $\tilde{h}^{\text {sat/mod}}$ to the set of tide gauges used for determining $\textbf{Q}_{\hat{\textbf{x}}^{\text {mod}}}$, we split the 137 records into two subsets (see Fig. 1): one subset contains 69 records, and the other 68.

**Fig. 3:**
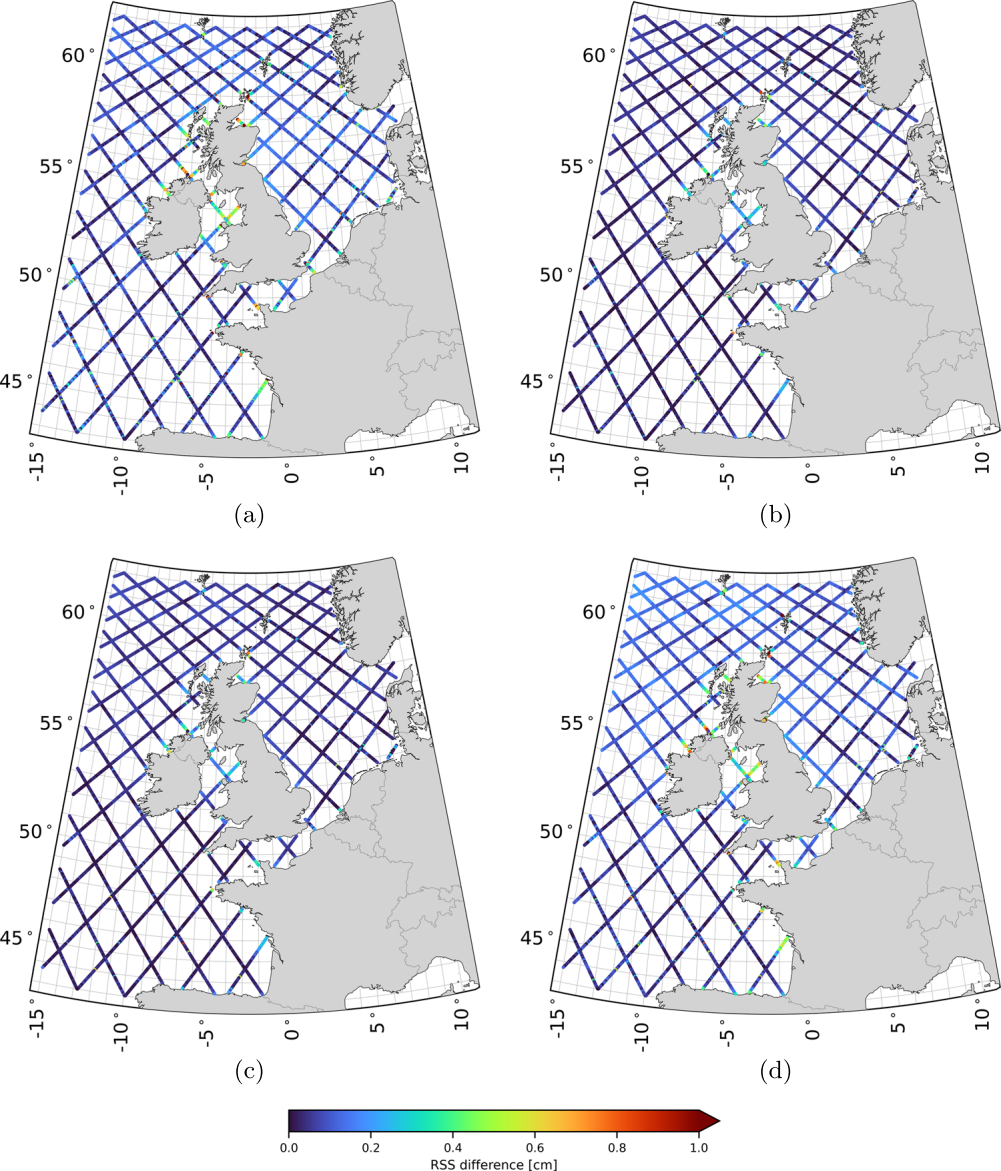
RSS values of the VDs between the tidal amplitudes and phases of all 93 constituents obtained with $\sigma$ is 4 cm (a), 5 cm (b), 7 cm (c), and 8 cm (d), and the ones obtained assuming $\sigma$ is 6 cm

## Results and discussion

Sect. 4.1 presents the computed along-track tidal product, along with the results of two sensitivity analyses. The first analysis quantifies the sensitivity of $\tilde{h}^{\text {sat/mod}}$ to the magnitude of $\sigma$ (see Eq. 4), while the second explores the sensitivity of $\tilde{h}^{\text {sat/mod}}$ to the set of tide gauges used to determine $\textbf{Q}_{\hat{\textbf{x}}^{\text {mod}}}$. In Sect. 4.2, we present and discuss product validation results. The final section assesses the product’s potential for future hydrodynamic model improvement by means of data assimilation.

### The along-track tidal product

Figure 2 shows the RSS values of the VDs between $\tilde{h}^{\text {sat/mod}}$ and $\hat{h}^{\text {mod}}$ (i.e., $\delta \tilde{h}^{\text {sat}}$) for all 93 constituents, as well as for specific categories of constituents (see Table 1). The RSS values for the other constituent categories listed in Table 1 are negligibly small. The maps show the following:

- The RSS values computed over all 93 VDs (Fig. 2a) can exceed 10 cm. The contribution of altimeter data to $\tilde{h}^{\text {sat/mod}}$, i.e. the ‘update’ to $\hat{h}^{\text {mod}}$ (see Eq. 5), is observed in both deep and shallow waters.
- The largest updates are in long-period (Fig. 2b) and semi-diurnal (Fig. 3b) constituents. Updates to long-period constituents are primarily concentrated in deep waters, particularly in the northwestern part of the domain, while those to semi-diurnal constituents are mainly seen in the shallow waters around Great Britain, especially in the English Channel and eastward between Edinburgh and Norwich, and in the German Bight. Also, in other areas, the updates to the semi-diurnal constituents reach 4 to 5 cm.
- The RSS values of the diurnal constituents (Fig. 2c) reach the 4 cm level in the Irish Sea and its northern entrance. Everywhere else, the magnitude is below 1 cm.
- Updates to the fourth- (Fig. 2e) and sixth-diurnal (Fig. 2f) constituents appear in the same regions. RSS values of the fourth-diurnal constituents reach magnitudes of 4 to 5 cm in the Bay of Biscay and the Hebrides waters. The RSS values of the sixth-diurnal constituents are lower, between 2 and 4 cm.

We consider RSS values with a magnitude of multiple centimeters to be substantial. However, whether the updates represent improvements remains to be verified. This verification is the focus of Sect. 4.2. One positive aspect is the spatial agreement in the RSS values, which is not only observed within the same pass but also across different passes. The method does not enforce similar adjustments at crossover points, making this spatial agreement noteworthy. In the discussion that follows, we interpret the results and assess whether the TPJ-derived updates to the DCSM-derived tidal constituents are explainable.

We primarily observe substantial updates to the long-period and semi-diurnal constituents. The estimated long-period constituents include, among others, the SA constituent, describing tidal variability at the annual frequency. It is common knowledge that tides are not the sole contributor to annual water level variability; winds, mean sea level pressure variations, and water density (baroclinic) variations also contribute. Since some of these processes (i.e., baroclinic water level variability) are not modeled by the DCSM, the residual water levels used in our analysis include these unmodeled effects. As a result, updates to the SA constituent likely reflect unmodeled annual water variability rather than improvements to the ‘tides’.

Regarding the semi-diurnal constituents, detailed analysis (not shown in this paper) has revealed that updates primarily affect the M2 constituent. This is expected, as the M2 constituent contributes substantially to tidal water level variability. The magnitude of the updates appears to correspond with the M2 amplitude itself. For example, large updates are observed in the shallow waters around Great Britain, where the tidal amplitude is large. In contrast, in the Dutch North Sea, tidal amplitudes are smaller due to the proximity of an amphidromic point, resulting in correspondingly smaller updates. However, it is important to note that the DCSM was specifically calibrated for water level predictions along the Dutch coast. As mentioned in Sect. 3.2, extra weight was assigned to Dutch coastal tide gauges in the cost function during calibration. This likely contributes to the smaller updates in Dutch waters, not just the low tidal amplitude, as the model was already well-tuned for this region.

Although the updates to the long-period and semi-diurnal constituents are the largest, this does not diminish the importance of updates to constituents belonging to other tidal groups. They all contribute to reconstructing the tide. We also observe spatial agreement in the RSS values for these constituents. Furthermore, it seems that, to a large extent, the updates are large where the amplitudes are large, which usually also leads to larger absolute errors for a model.

#### Sensitivity of $\tilde{h}^{\text {sat/mod}}$ to the magnitude of $\sigma$

To evaluate the sensitivity of $\tilde{h}^{\text {sat/mod}}$ to $\sigma$ (i.e., the standard deviation of the uncertainty in the TPJ-derived sea surface heights plus the uncertainty due to unmodeled or mismodeled nontidal water level variability that is used to define $\textbf{Q}_{{h}_{\text {total}}^{\text {sat}}}$ (see Eq. 4)), we computed the RSS values of the VDs between the tidal amplitudes and phases of all 93 constituents obtained using a varying $\sigma$ and the ones obtained assuming $\sigma$ is 6 cm. In our analysis, $\sigma$ increases from 4 to 8 cm in steps of 1 cm. Figure 3 shows the maps of RSS values. The results indicate:

- For all $\sigma$ values considered, the RSS values are well below 1 cm, with values below 0.5 cm for $\sigma$ values of 5 cm and 7 cm.
- Besides some minor, random deviations, the RSS values show some systematic behavior. Areas exhibiting heightened sensitivity include the Irish Sea and its northern entrance, the Bristol Channel, the English Channel near the French/Belgian coast, the coastal area adjacent to the Bay of Biscay, and the Moray Firth in northern Scotland.

The assumed value of $\sigma = 6$ cm, used to compute the along-track tidal product, is robust to small changes in $\sigma$ (1–2 cm). We consider this positive since it lacks rigorous justification. This also applies to the assumption that the value is uniform across the entire model domain. Uncertainties due to unmodeled or mismodeled nontidal water levels are expected to vary spatially and temporally. Additionally, correlations, which certainly exist, have been ignored. Developing a noise model based on which a realistic approximation of $\textbf{Q}_{{h}_{\text {total}}^{\text {sat}}}$ can be formulated would be an improvement, though it is beyond the scope of this research.

**Fig. 4:**
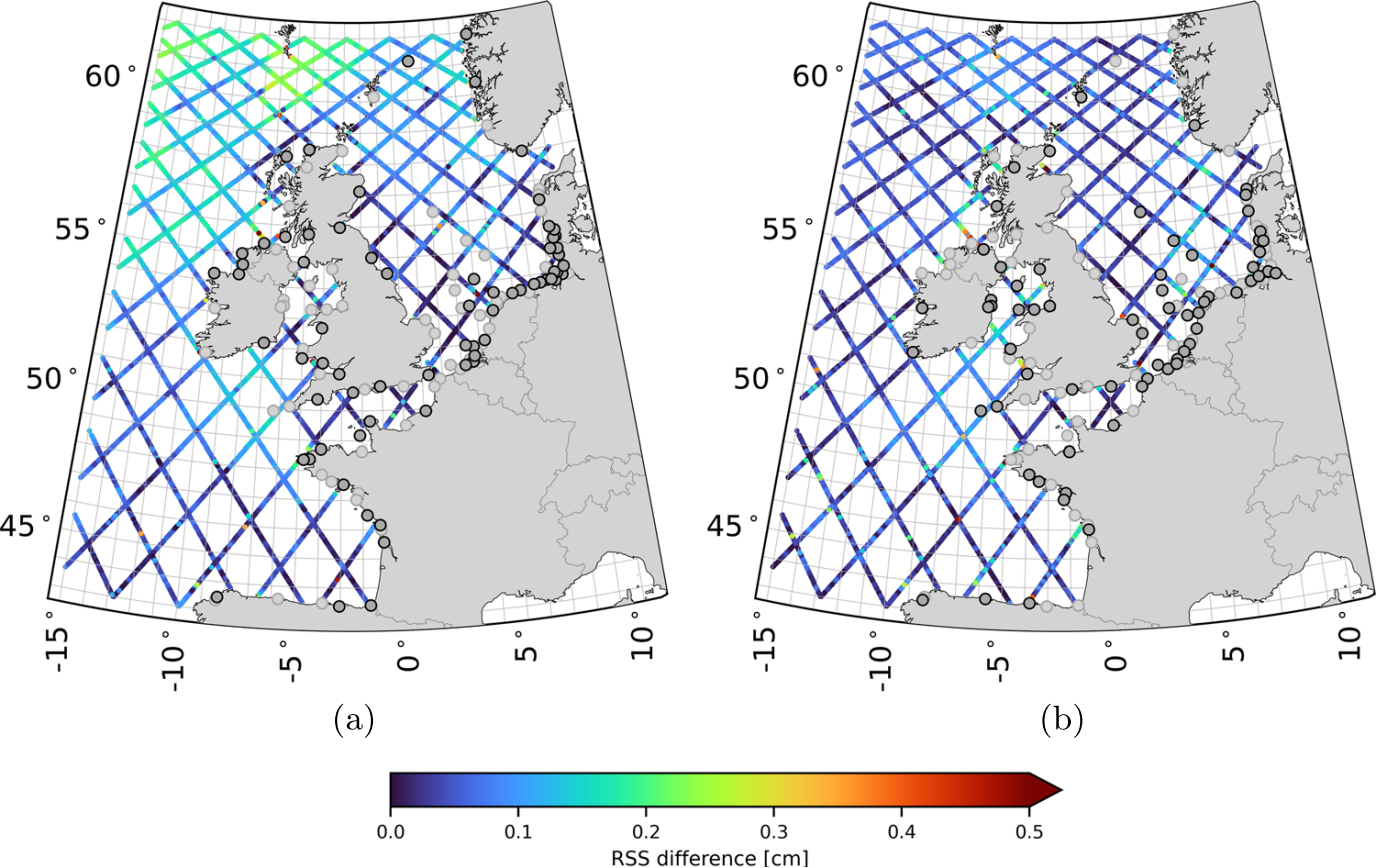
RSS values of the vector differences (VDs) between tidal amplitudes and phases for all 93 constituents, comparing results from two alternative realizations of $\textbf{Q}_{\hat{\textbf{x}}^{\text {mod}}}$ (using non-overlapping subsets of the 137 tide gauges) with those obtained from a $\textbf{Q}_{\hat{\textbf{x}}^{\text {mod}}}$ computed using the full set of 137 tide gauges. Panels (a) and (b) show the results for each alternative realization

#### Sensitivity of $\tilde{h}^{\text {sat/mod}}$ to the set of tide gauges used to determine $\textbf{Q}_{\hat{\textbf{x}}^{\text {mod}}}$

To assess the sensitivity of $\tilde{h}^{\text {sat/mod}}$ to the set of tide gauges used to determine $\textbf{Q}_{\hat{\textbf{x}}^{\text {mod}}}$, we compared the estimated amplitudes and phases to the ones obtained with two alternative realizations of $\textbf{Q}_{\hat{\textbf{x}}^{\text {mod}}}$. Each is based on a subset of the 137 tide gauges used before. The two subsets do not overlap and have a comparable geographic distribution. The results, summarized by the RSS differences for all 93 constituents, are shown in Fig. 4. From the maps, we observe:

- The RSS values obtained when using the two alternative realizations of $\textbf{Q}_{\hat{\textbf{x}}^{\text {mod}}}$ are, except for some outliers, all below 0.3 cm.
- The RSS values show regional differences. In Fig. 4a the values are somewhat larger, notably in deep waters.

In the calculation of $\textbf{Q}_{\hat{\textbf{x}}^{\text {mod}}}$, we utilized *all* 137 tide gauge records that met the criteria outlined in Sect. 3.5. The analysis presented here demonstrates that halving this number has little impact on the results. We consider this as a positive outcome. However, this finding does not fully address the robustness of the results, especially considering the non-uniform distribution of tide gauges across the model domain, with most records concentrated along the Dutch coast.

Furthermore, it is important to note that there are $$\left( {\begin{array}{c}137\\ 68\end{array}}\right) $$ possible ways to split the set of 137 tide gauge records into two subsets. We only examined two of these possibilities, both of which had comparable geographical distributions. Whether the estimated amplitudes and phases remain the same when the distribution is no longer comparable (e.g., if one subset contained only tide gauges outside the North Sea) is an open question. The analysis also does not determine the minimum number of tide gauge records required to reliably determine $\textbf{Q}_{\hat{\textbf{x}}^{\text {mod}}}$. Additionally, ignoring the spatial correlations makes the method and the interpretation simpler. However, it may be sub-optimal. Now, the method just weighs the errors from the model and observations per constituent.

Overall, there is ample room for further research. The extent to which our understanding of the tides has improved will be assessed in the validation presented in Sect. 4.2. If so, this discussion highlights that the method’s full potential has yet to be fully realized.

**Fig. 5:**
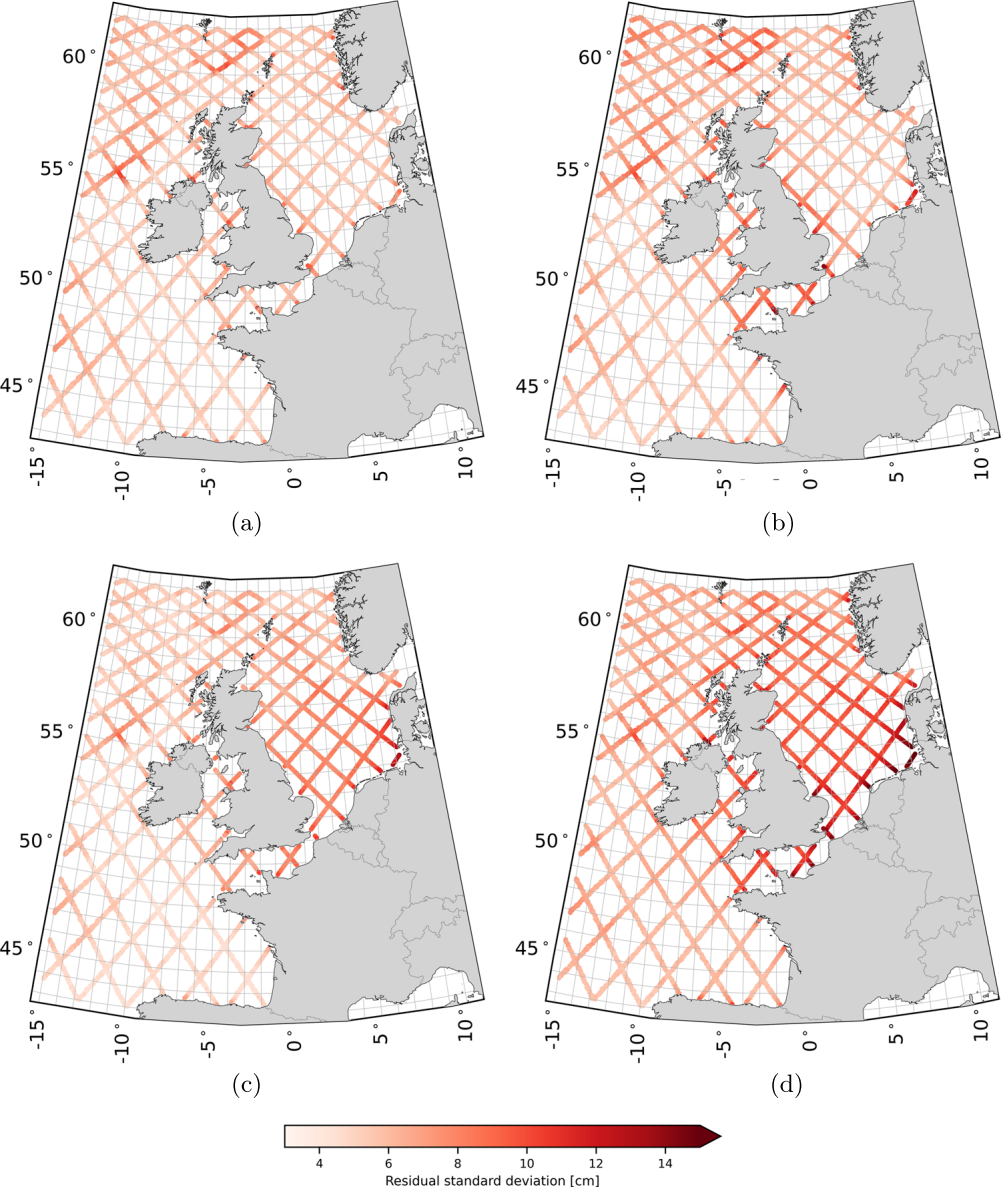
SDs of the residual water level time series corresponding to set $\xi ^{(1)}$ (a), $\xi ^{(2)}$ (b), $\zeta ^{(1)}$ (c), and $\zeta ^{(2)}$ (d). For the definitions of the sets we refer to Table 2

### Validation

Figure 5 shows the maps of the SDs of the residual water level time series corresponding to set $\xi ^{(1)}$, $\xi ^{(2)}$, $\zeta ^{(1)}$, and $\zeta ^{(2)}$ (see Table 2 for the definitions of the sets). The maps for sets $\varsigma ^{(1)}$, $\varsigma ^{(2)}$, and $\varsigma ^{(3)}$ are not included because they are indistinguishable from one another. Summary statistics for all seven sets defined in Table 2, broken down for deep and shallow waters, are presented in Table 3. The results show the following:

- With the exception of the maximum SD in deep waters, all summary statistics for $\xi ^{(1)}$ are lower than those for $\xi ^{(2)}$. In deep waters, the differences range from 2 mm (maximum) to 1 cm ($95\%$). In shallow waters, the differences range from 5 mm (minimum) to 4.8 cm (maximum).
- In the areas where we observed the largest updates to the semi-diurnal constituents, i.e. in the shallow waters around Great Britain (notably in the English Channel and in the east between Edinburgh and Norwich) and in the German Bight (see Fig. 2d), the SDs reduced from 11 cm to 5 cm (cf Fig. 5a and b).
- In shallow waters ($<200$ m), the SDs are lowest for $\xi ^{(1)}$. In terms of the median, the value is 6.0 cm compared to 6.6 ($\xi ^{(2)}$), 7.5 ($\zeta ^{(1)}$), and 10.1 cm ($\zeta ^{(2)}$).
- In deep waters ($>200$ m), the SDs are lowest for $\zeta ^{(1)}$. In terms of the median, the value is 5.5 cm compared to 6.2 ($\xi ^{(1)}$), 6.8 ($\xi ^{(2)}$), and 7.0 cm ($\zeta ^{(2)}$). Compared to the SDs of $\xi ^{(2)}$, the SDs of $\xi ^{(1)}$ are lower.
- The SDs of $\xi ^{(1)}$ are the most homogeneous across the entire domain; the median of all SDs in deep waters is 6.2 cm, while in shallow waters the median is 6.0 cm. For $\xi ^{(2)}$, the difference in median values is the same, but the values are higher. For $\zeta ^{(1)}$ and $\zeta ^{(2)}$, the median values of the SDs in deep and shallow waters differ by multiple centimeters. In shallow waters, $90\%$ of all SDs are between 5.1 and 8.0 cm. For $\xi ^{(2)}$, $\zeta ^{(1)}$, and $\zeta ^{(2)}$ the range is at least 1.9 cm larger. Compared to the SDs of $\xi ^{(2)}$, we observe that the SDs of $\xi ^{(1)}$ in the southern North Sea, the English Channel, and the waters between Ireland and the UK are comparable to the values in the central North Sea. Compared to the SDs of $\zeta ^{(1)}$ and $\zeta ^{(2)}$, we do not observe a distinct behavior in deep and shallow and/or coastal waters.
- The SDs of $\varsigma ^{(1)}$, $\varsigma ^{(2)}$, and $\varsigma ^{(3)}$ are comparable, both in deep and shallow waters. The median of all SDs only varies at the millimeter level.

**Table 3:** Summary statistics of the SDs of the residual water level time series, in centimeters, for the seven sets defined in Table 2

Domain	Set	mean	SD	min	5%	25%	50%	75%	95%	max
deep	$\xi ^{(1)}$	6.4	1.1	4.5	5.0	5.6	6.2	6.9	8.7	11.9
($>200$ m)	$\xi ^{(2)}$	7.0	1.4	4.7	5.3	5.9	6.8	7.8	9.7	11.7
	$\zeta ^{(1)}$	**5**.**8**	**1**.**0**	**4**.**2**	**4**.**7**	**5**.**1**	**5**.**5**	**6**.**3**	**7**.**7**	**10**.**3**
	$\zeta ^{(2)}$	7.3	**1**.**0**	5.4	6.1	6.6	7.0	7.7	9.4	11.6
	$\varsigma ^{(1)}$	12.9	1.9	8.5	9.7	11.2	13.3	14.4	15.5	17.9
	$\varsigma ^{(2)}$	13.1	2.0	8.2	9.9	11.2	13.5	14.7	15.8	17.8
	$\varsigma ^{(3)}$	13.0	1.8	8.7	10.0	11.3	13.3	14.5	15.4	17.2
shallow	$\xi ^{(1)}$	**6**.**2**	**1**.**0**	4.6	5.1	**5**.**6**	**6**.**0**	**6**.**4**	**8**.**0**	**14**.**6**
($<200$ m)	$\xi ^{(2)}$	7.2	1.6	5.1	5.7	6.2	6.6	7.8	10.5	19.4
	$\zeta ^{(1)}$	7.6	1.8	**4**.**4**	**5**.**0**	6.2	7.5	8.9	10.3	16.2
	$\zeta ^{(2)}$	10.1	2.2	5.9	7.0	8.7	10.1	11.3	13.9	22.5
	$\varsigma ^{(1)}$	14.1	2.2	10.0	11.5	12.7	13.6	15.1	17.8	25.7
	$\varsigma ^{(2)}$	14.4	2.4	10.2	11.7	12.8	13.9	15.7	18.7	26.0
	$\varsigma ^{(3)}$	14.4	2.4	10.4	11.7	12.8	13.9	15.5	19.1	27.5

Lowest values are in bold

**Table 4:** Summary of cross-over RSS of the vector differences and posterior values, in centimeters, for different harmonic constituents sets

Category	All	Shallow ($<200m$)	Deep ($>200m$)	Posterior
All	1.48	1.54	1.46	2.50
Long-period	0.33	0.34	0.32	0.53
Diurnal	0.41	0.47	0.37	0.69
Semi-diurnal	0.87	0.93	0.84	1.32
Fourth-diurnal	0.87	0.90	0.86	1.08
Sixth-diurnal	0.48	0.46	0.52	1.01

The lower SDs of $\xi ^{(1)}$ compared to $\xi ^{(2)}$ can be interpreted as an improvement in the tidal constituents. This conclusion is justified by the strict separation between the data used to estimate the constituents and the data used for validation, ensured by our *K*-fold cross-validation strategy. The improvements are substantial and visible over the entire domain. The largest improvements in the shallow waters are located in physically plausible regions, such as the English Channel and the southern North Sea. It is important to note that further improvements are possible. As mentioned in Sect. 1, some simplifications were employed in our approach. For instance, we used a set of tidal constituents defined for Dutch coastal waters across the entire DCSM domain. Customizing the tidal constituent set for various sub-regions could potentially reduce residual tidal water levels further, leading to even lower SDs.

Indeed, the improvements are evident only when the complete tide-surge water levels are removed before computing the SDs. When only the tides are removed, the SDs remain comparable in both deep and shallow waters. This suggests that the variability caused by surge and tide-surge interactions is larger than the magnitude of the residual tidal errors, effectively masking any improvements in tidal water levels. The more homogeneous SDs of $\xi ^{(1)}$ in shallow waters, compared to those of $\xi ^{(2)}$, support the conclusion of Guarneri et al. (2023) that the reported lower accuracy of estimated tidal constituents in shelf and coastal waters (e.g., Stammer et al. 2014) is due to the omission of nonlinear tide-surge interactions when correcting the observed water levels.

However, the results do not allow us to make definitive statements about the quality of our product compared to X-TRACK. The lower SDs of $\xi ^{(1)}$ in shallow waters are largely due to the inclusion of nonlinear tide-surge interactions. In contrast, these interactions are not taken into account in the calculation of $\zeta ^{(1)}$. In deep waters, where nonlinear tide-surge interactions do not occur, $\zeta ^{(1)}$ shows lower SDs. This is likely due to the better performance of X-TRACK in deep waters. However, this is not entirely certain, as different surge correction are also applied. On the other hand, comparing $\xi ^{(2)}$ and $\zeta ^{(1)}$ indicates that using the DCSM model as a prior leads to a much larger reduction in variance. This benefits the least-squares estimation and likely improves tide accuracy.

**Fig. 6:**
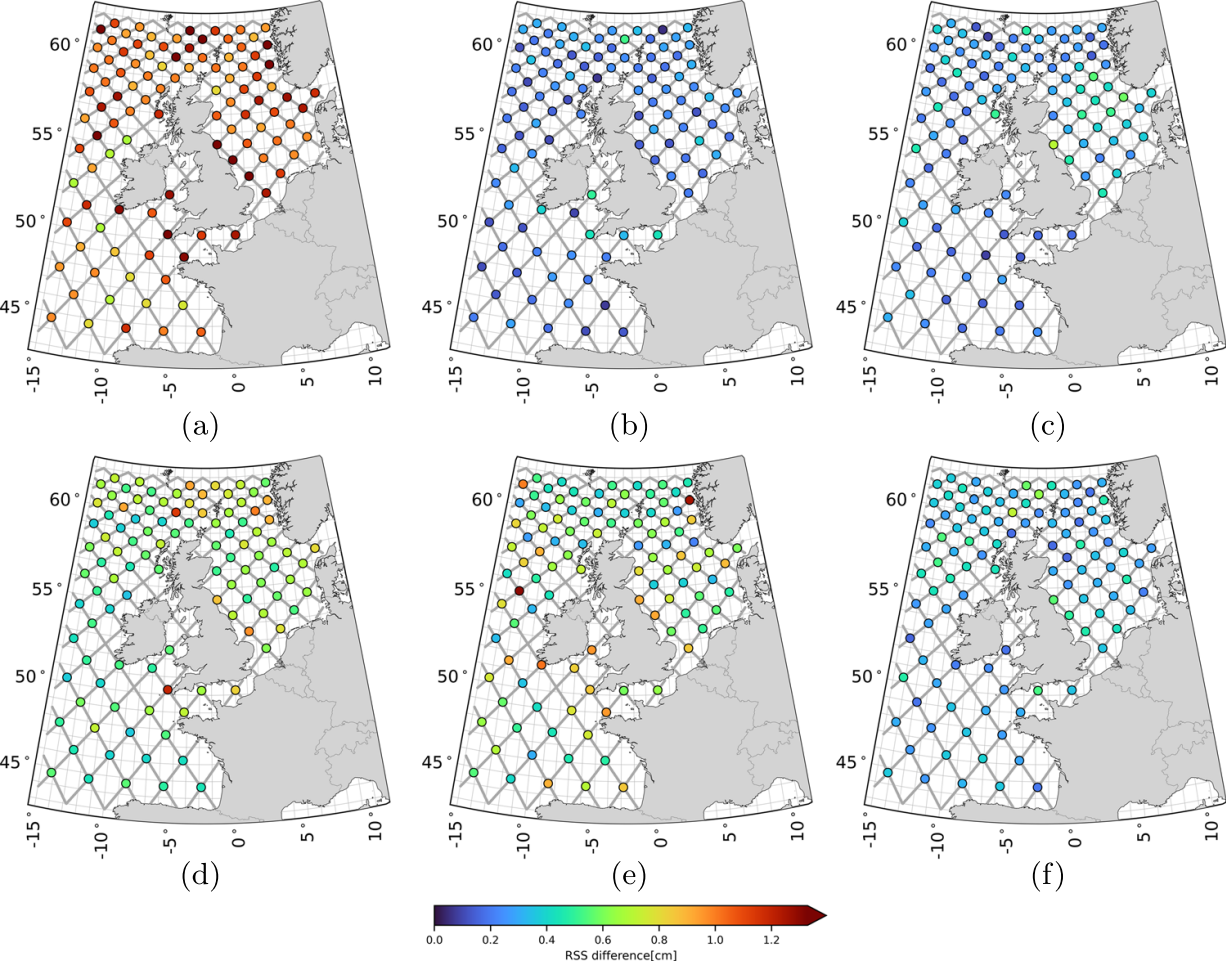
Cross-over RSS values of the VDs for all 93 constituents (a), the long-period constituents (b), the diurnal constituents (c), the semi-diurnal constituents (d), the fourth-diurnal constituents (e), and the sixth-diurnal constituents (f)

The SDs were calculated over a 24 years time series. We did not investigate whether the SD magnitudes vary over this period. Probably this is the case. For example, Slobbe et al. (2018) has shown that an earlier version of the DCSM performed worse in representing the monthly mean water levels in winter than in summer. However, any potential variations in the magnitude of the SDs over time are unlikely to reflect changes in the tide or its model representation. While tidal changes do occur in the area of interest (e.g., Bij de Vaate et al. 2022), these changes are at the millimeter level and can be safely ignored for the purposes of this study.

To gain further insight into the performance of our product, we assessed the differences between the estimated amplitudes and phases at crossover points, which are where the ascending and descending tracks of the satellite intersect. Since the updates for both tracks are largely independent and of similar size, we can use the VD as a measure of accuracy. Table 4 and Fig. 6 show the accumulated statistics for tidal bands, as in Fig. 2. The average total tide error is estimated at an RSS vector difference of 1.48 cm, with the semi-diurnal and fourth-diurnal tides being the largest contributors (both 0.87 cm). As expected, the errors are slightly larger than those in deep water, but the differences are small. The last column of Table 4 also shows the error estimates from the weighted least-squares. These estimates are slightly larger (2.50 cm total vs. 1.48 cm). We attribute this difference mainly to the value of the SD for the measurement errors (6.0 cm); decreasing this value would lower the estimated errors, even though the impact on the estimates is small. The spatial distribution of the errors in Fig. 6 is quite homogeneous, but crossovers near the coast exhibit larger errors in several cases. This is potentially due to the accuracy of the altimeter data or the larger amplitudes and complexity of tides near the coast.

**Fig. 7:**
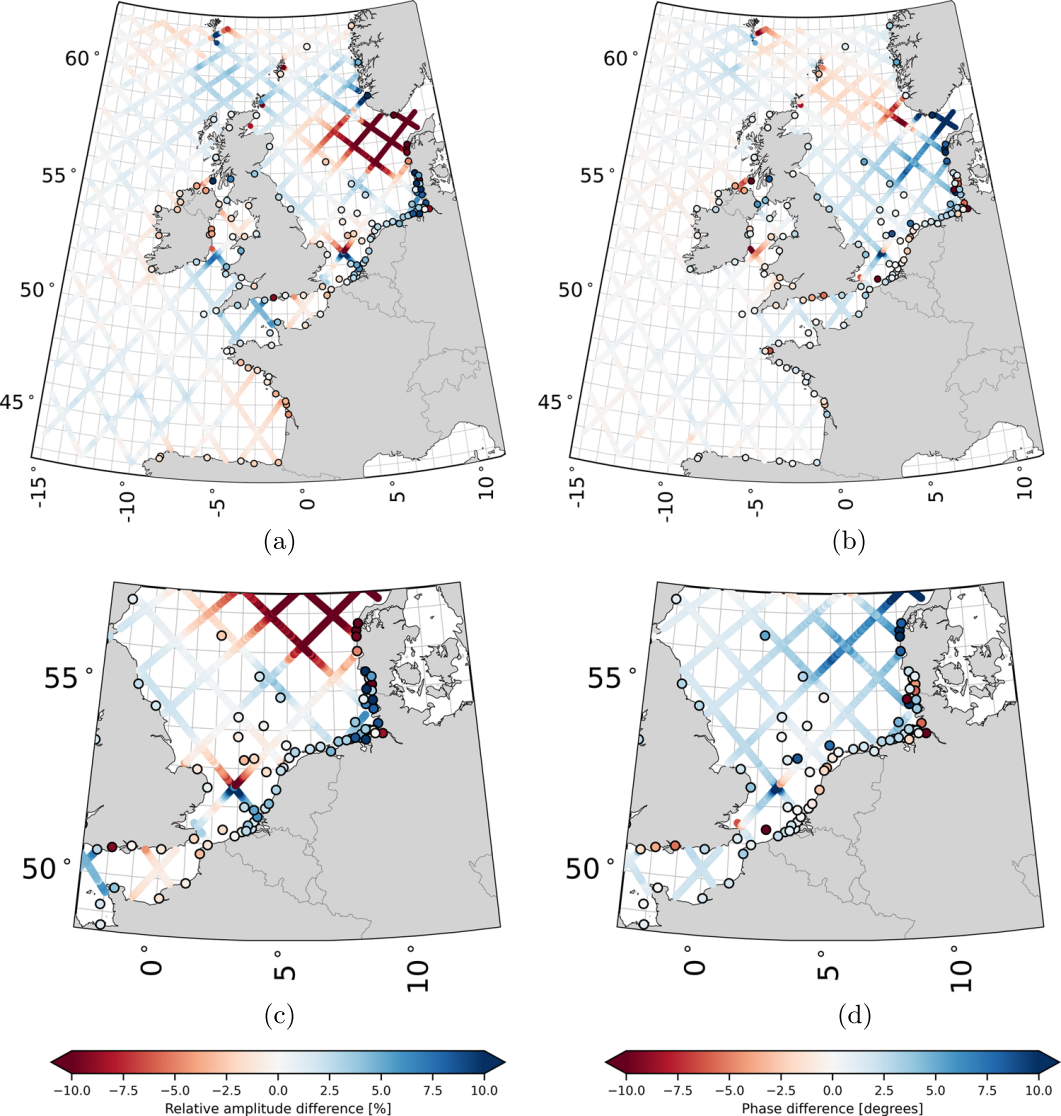
Panels (a) and (b) show M2 difference patterns for amplitude and phase, respectively, where amplitude differences are represented as relative differences (%) and phase differences as absolute differences in degrees. Along each track, the differences between the product and model are shown, while at station locations, the differences between station data and the model are displayed. Similar patterns between the along-track and station differences suggest that the along-track product effectively captures deficiencies in the model’s tidal representation, supporting its utility for model calibration. Panels (c) and (d) provide zoomed-in views of panels (a) and (b), respectively. Note that all points with an amplitude smaller than 2 cm have been removed

### The potential of the along-track tidal product for improving the DCSM

Figure 7 shows the residual M2 tidal amplitudes and phases estimated from the altimeter data, along with the DCSM errors in M2 tidal amplitude and phases at the tide gauge locations. All values are normalized by the corresponding local DCSM-derived amplitudes and phases. The results corresponding to the constituents S2, K1, and O1 are included in Appendix A. From the figures, we observe:

- Overall, there is a good agreement between values at the altimeter data locations and those at the tide gauge locations. For M2, some notable exceptions for the phase are observed in the German Bight, the Irish Sea, and the English Channel.For O1, the correction direction aligns with nearby stations in the Irish Sea but shows lower amplitudes. For K1, there is a slight phase correction disagreement in the North Sea.
- For M2, S2, and K1, the largest values, up to 20 %, are observed on the shelf. Notably, in the waters south of Norway, the high M2 relative differences result from smaller modeled M2 amplitudes used for the normalization.
- For K1 and O1, the maps reveal a gap south of Norway. At all points within this gap, the amplitude was smaller than the 2 cm threshold used to clean the data. When the amplitude is small, both the amplitude ratio and phase become unreliable.
- The values at the altimeter data locations show spatial agreement.

**Fig. 8:**
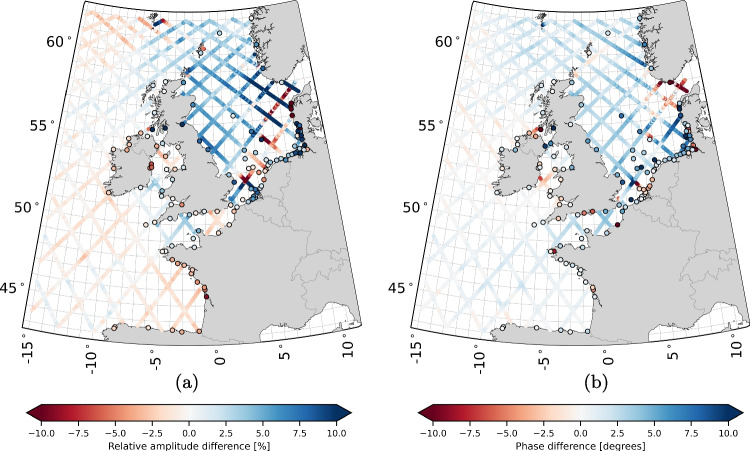
The caption is the same as in Fig. 7. This figure shows the difference patterns for amplitude and phase for the S2 constituent

**Fig. 9:**
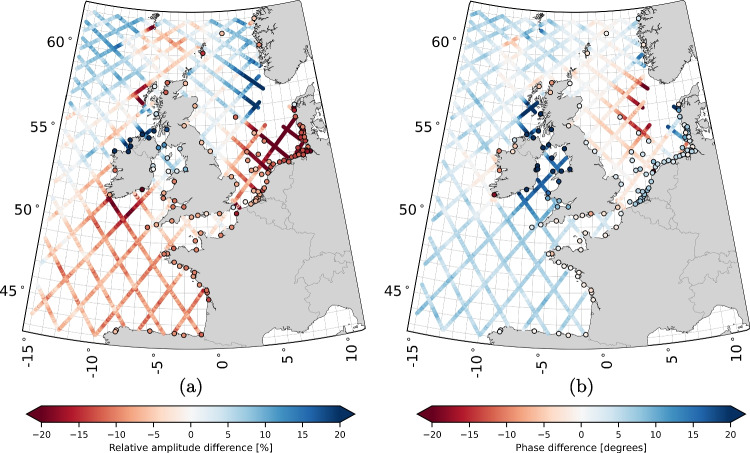
The caption is the same as in Fig. 7. This figure shows the difference patterns for amplitude and phase for the K1 constituent

**Fig. 10:**
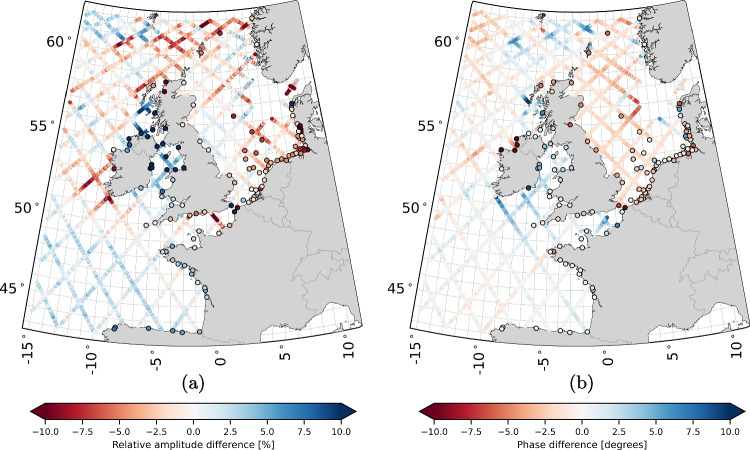
The caption is the same as in Fig. 7. This figure shows the difference patterns for amplitude and phase for the O1 constituent

If we treat the amplitudes and phases derived from tide gauge records as ground truth, the differences between these and the DCSM-derived values represent errors in the DCSM. Despite the DCSM being calibrated with tide gauge data, these errors are substantial. As noted in Sect. 3.2, the calibration prioritized improving the DCSM’s accuracy along the Dutch coast, with extra weight given to Dutch coastal tide gauges in the cost function. Accordingly, Fig. 7 and Figs. 8, 9 and 10 show that the largest errors occur outside Dutch waters. In addition, some errors may stem from our reliance on equilibrium tide theory for the nodal correction (see Sect. 2.2) and the fact that the model calibration was based on tide gauge data from 2017. In contrast, the amplitudes and phases shown here are derived from at least five consecutive years of tide gauge data (1993–2017).

Given that the same model serves as the background for our along-track tidal product, the residual amplitudes and phases should, assuming accuracy, align with those observed at the tide gauge stations. The results confirm this, offering further validation of our product. For the intended application of this along-track tidal product, the results particularly highlight its potential to enhance the DCSM, especially when both datasets are used together. It is important to note that discrepancies in regions like the German Bight, Irish Sea, and English Channel do not necessarily imply inaccuracies in our product. The German Bight, for example, is a complex region with strong spatial variations over short distances, complicating the interpretation of tidal errors.

## Conclusions and recommendations

This study builds on the findings of Guarneri et al. (2023), where we found that ignoring nonlinear tide-surge interactions when removing tide and surge estimates from satellite altimeter-derived water levels prior to tidal harmonic analysis significantly reduces the accuracy of altimeter-derived tidal constituents in shelf and coastal waters. The primary objective here was to develop a procedure for generating an along-track tidal product from satellite altimeter data that exploits a 2D hydrodynamic model accounting for these nonlinear interactions. We demonstrated this procedure using TOPEX/Poseidon and Jason (TPJ) altimeter data for the northwest European continental shelf, validated the obtained along-track tidal product, and assessed its potential for future hydrodynamic model calibration through data assimilation.

The proposed method treats the model-derived tidal constituents as stochastic, allowing altimeter data to update the values based on relative precision. The resulting tidal product is a combined altimeter- and model-derived product, with updates to tidal constituents reaching over 10 cm in the root-sum-square (RSS) of vector differences for 93 constituents. Significant updates were observed for long-period and semi-diurnal constituents, particularly in deep waters and shallow regions around Great Britain and the German Bight. Diurnal and higher-order constituents also showed notable improvements in various areas. The product demonstrated robustness against the assumed level of uncertainty of the altimeter-derived water levels, with RSS variations staying below 0.5 cm. It also showed consistency across different tide gauge selections used for calculating the variance-covariance matrix of the model-derived tidal constituents, with RSS values under 0.3 cm.

Validation using residual water level standard deviations (SDs) indicated that our demonstration product provides lowers the values from 11 cm to 5 cm in the shallow waters around Great Britain and in the German Bight. In deep waters ($>200$ m), the median standard deviation decreased from 6.8 cm to 6.2 cm. When compared to state-of-the-art ocean tide (i.e., X-TRACK) and surge (i.e., DAC) corrections from publicly available models, our method outperformed them in shallow waters (median standard deviation of 6.0 cm versus 7.5 cm), though the alternative products performed better in deep waters (median standard deviation of 5.5 cm versus 6.2 cm). An analysis of the vector differences of the tidal constituents provides a more direct estimate of the accuracy of 1.5cm RSS over all tidal constituents. This analysis confirms that the errors on the shelf are comparable to those in deep water. We acknowledge that comparisons in shallow waters are complicated, as alternative products do not account for nonlinear tide-surge interactions. Finally, the new product showed potential to improve the hydrodynamic model used in this study, demonstrating strong agreement between model-derived and altimeter-estimated amplitudes and phases for four main constituents at tide gauge locations.

These findings reinforce the conclusion from (Guarneri et al. 2023) regarding the importance of accounting for nonlinear tide-surge interactions when estimating tides from satellite altimeter data in shallow waters. This is significant because it suggests that altimeter data holds more potential than currently utilized. Doing so may improve the connection with the tide gauges, which are predominantly located along the coast.

However, this study did not explore the applicability of our method in regions without the extensive tidal observation network that exists in the North Sea. In such regions, the number of tidal constituents that can be reliably estimated may be limited, and alternative strategies might be needed to evaluate the model’s uncertainty in representing tides. Ideally, the installation of tide gauges in key locations would help address this limitation.

Future research will address the simplifications employed in our approach, including the use of fixed set of tidal constituents for the entire domain covered by the hydrodynamic model and the reliance on equilibrium tide theory in computing the nodal corrections. Moreover, future research could investigate the role of the non-diagonal components in the variance-covariance matrix of the model-derived tidal constituents and assess the impact of regionalizing $\textbf{Q}_{\hat{\textbf{x}}^{\text {mod}}}$. This would enable a deeper exploration of the information content of satellite radar altimeter data for estimating tidal constituents in shallow waters. Another opportunity for future research would involve applying this method using a three-dimensional model, such as the 3D DCSM-FM (Zijl et al. 2022), which can account for baroclinic processes and their nonlinearities. In the current study, part of the baroclinic signal persists during the tidal inversion and is treated as part of the stochastic model. We anticipate further improvements in accuracy as nontidal signals are reduced using a 3D model.

## Data Availability

No datasets were generated or analysed during the current study.
